# Annurca apple (M. pumila Miller cv Annurca) extracts act against stress and ageing in *S. cerevisiae* yeast cells

**DOI:** 10.1186/s12906-017-1666-7

**Published:** 2017-04-05

**Authors:** Mariarita Stirpe, Vanessa Palermo, Michele Maria Bianchi, Romano Silvestri, Claudio Falcone, Giancarlo Tenore, Ettore Novellino, Cristina Mazzoni

**Affiliations:** 1 Pasteur Institute-Cenci Bolognetti Foundation, Department of Biology and Biotechnology ‘Charles Darwin’, Sapienza University of Rome, Piazzale Aldo Moro 5, 00185 Rome, Italy; 2 Pasteur Institute-Cenci Bolognetti Foundation, Dipartimento di Chimica e Tecnologie del Farmaco, Sapienza University of Rome, Piazzale Aldo Moro 5, I-00185 Rome, Italy; 3 Department of Pharmacy, University of Naples “Federico II”, Naples, Italy

**Keywords:** Apoptosis, Fruit, Polyphenols, Anti-oxidant, ROS, Mitochondria

## Abstract

**Background:**

During the past years, a number of studies have demonstrated the positive effect of apple on ageing and different diseases such as cancer, degenerative and cardiovascular diseases.

The unicellular yeast Saccharomyces cerevisiae represents a simple eukaryotic model to study the effects of different compounds on lifespan. We previously demonstrated that apple extracts have anti-ageing effects in this organism because of their antioxidant properties.

In particular, the effect is related to the presence in this fruit of polyphenols, which give a large contribution to the antioxidant activity of apples.

**Methods:**

We we used a clonogenic assay to assess the viability and the resistance to oxidative stress of *S. cerevisiae* cells in the presence of Annurca apple extracts.

The production of ROS and the aberrant morphology of nuclei were detected by cell staining with the fluorescent dies Dihydrorhodamine 123 and DAPI, respectively. Mitochondrial morphology was analyzed by following the localization of the mito-GFP protein into the mitochondrial matrix.

**Results:**

In this study, we show that apple extracts can increase yeast lifespan, reduce the levels of reactive oxygen species and cell sensitivity to oxidative stress, and prevent nuclei and mitochondria fragmentation protecting cells from regulated cell death.

**Conclusions:**

In this paper, by using the yeast *S. cerevisiae* as a model, we have demonstrated that Annurca extracts possess antioxidant properties thanks to which the extracts can reduce the intracellular ROS levels and have anti-apoptotic functions thus prolonging cell lifespan.

These results contribute to knowledge on the effects of natural compounds on ageing and support the use of yeast as a model organism for the development of simple tests to assess the effectiveness of bioactive substances from natural sources.

## Background

Ageing, defined as the progressive loss of function in all constituents of living cells, is a complex biological process involving many factors at the same time. Studies on humans concerning the deciphering of ageing mechanisms are not easy and the duration of ageing is one of the main limiting factors. For these reasons, model systems, such as yeast, have become particularly useful to study the mechanism of ageing thanks to their simple handling and because they share a large number of genes and cellular pathways with humans.

Studies on the relationships between diet and ageing are growing and it was demonstrated that calorie restriction [1] and some antioxidants [2–4] can extend lifespan in yeast and other model organisms [5, 6].

Fruit and vegetable consumption has been related with improving health thanks to the supply of phytochemicals. The phytochemicals show a wide range of biological activities which may contribute to health beneficial effect against diseases including cancer, cardiovascular diseases, diabetes, pulmonary disorders, Alzheimer and other degenerative diseases [7].

In particular, the healthy effects of apples and their derivatives, such as juice, cider, vinegar and distillates have gained the attention of the scientific world and are currently studied in different research groups.

The most studied compounds found in apples are polyphenols, which are present in high levels ranging from 0.01 to 1% of the fresh weight of this fruit. Polyphenols, including chlorogenic acid, phloretin, proanthocyanidin B2, epicatechin, chatechin and rutin, are responsible for the main antioxidant activity of apples [8].

Since the mechanisms underlying the anti-ageing properties of apples still need to be clarified, in this work we studied the effects of apple on some aspects of yeast cell ageing.

Yeast has been employed with success in the determination of the cellular effects on ageing and cell death. Some phytochemicals and substances, such as *Haberlea rhodopensis* extracts [9] and carnitines [2], promote cell vitality while, on the opposite, peppermint [10] and cooking oils [11] exert toxic effects.

In a previous work, we demonstrated the positive effect of Golden Delicious apple components in reducing ROS levels, in preventing ageing and cell death in the yeast pro-apoptotic mutant *Kllsm4Δ1* [3]. Cells of this strain, that express a truncated form of the essential protein Lsm4p involved in mRNA degradation, are still viable but show premature ageing and apoptotic death following a metacaspase-dependent pathway [12–16].

In this paper, we focused on a different sort of apple, namely Melannurca Campana, which is a small an tasty apple grown in the Campania region of Italy for at least two millennia.

This fruit, which has gained recognition of Protected Geographical Indication (PGI), is widely consumed by the Italian population and is produced in a quantity of about 60,000 tons *per* year in the average.

Melannurca Campana possesses healthy and nutritious properties for the high content of vitamins (B1, B2, PP and C) and minerals (potassium, iron, phosphorus, manganese), The apple also exerts gastroprotective action due to the richness in phenolic compounds, in particular procyanidins, in much higher concentration than any other apple.

In addition, Melannurca seems to reduce total cholesterol and increase expression of HDL [17, 18].

In the present work, we describe the positive effect of extracts from Melannurca on the longevity of the *Kllsm4Δ1* cells. Moreover, we demonstrate that the same extracts, beside the reduction of ROS levels and cell sensitivity to oxidative stress, can also lower nuclear fragmentation and mitochondrial morphology defects in yeast cells under stress.

## Methods

### Strains and yeast growth

We used the *S. cerevisiae* strain MCY4/*Kllsm4Δ1* (*Mat α, ade1-101, his3-D1, trp1-289, ura3, LEU2- GAL1-SDB23, pRS313/Kllsm4Δ1*) [15]. Cells were grown at 28 °C in SD (0.67% yeast nitrogen base without amino acids, 2% glucose and auxotrophic requirements as needed). Solid media were supplemented with 2% Bactoagar (Difco, Detroit, MI, USA).

### Preparation of apple extracts

Extracts from Melannurca Campana apples were used for the production of dietary supplement capsules containing 63% procyanidin, 20% phloridzin and added with maltodextrins. The dietary supplement was produced by Fitolife s.r.l. laboratory (Naples, Italy) and the quality of the product was certified by the Department of Pharmacy of Federico II°-University of Naples (Italy).

### Cell viability

The determination of chronological lifespan was done by the method of microcolonies as already described [19]. Briefly, cell suspensions (5 *μ*L) containing approximately 6 *·* 10^6^ cells/ml were poured on a thin layer of YPD agar on a microscope slide. A cover slip was placed over the samples and, after 24 h, viable and unviable cells were scored on the basis of their ability to form microcolonies. Cells were grown at 28 *°*C in SD minimal medium supplemented with 20 μg/ml of the appropriate nutritional requirements according to the genotype of the strains. For the viability spot assay, cell suspensions at the concentration of 10^7^ cells/ml were transferred into microtiter plates, serially ten-fold diluted and spotted onto YP plates (1% yeast extract, 2% peptone) supplemented with 2% glucose (YPD). Plates were incubated at 28 *°*C for 3 days before recording.

### H_2_O_2_ sensitivity

To determine the sensitivity to hydrogen peroxide, cells growing exponentially were exposed to 0.8, 1.2, and 3 mM H_2_O_2_ at 28 *°*C for 4 h. Cell viability was determined by counting the the number of microcolonies formed.

### Fluorescence microscopy

Dihydrorhodamine 123 (Sigma) was added at the concentration of 5 μg/ml to cell cultures. After 3 h of incubation cells were visualized under the microscope using a rhodamine optical filter [20].

For DAPI staining, following standard yeast protocols, exponentially growing cells were harvested, fixed in 70% ethanol, stained with 1 μg/ml DAPI solution and observed by fluorescence microscopy. For the analysis of mitochondrial morphology, cells were transformed with plasmid pYX232-mtGFP [21] that targets the Green Fluorescent Protein (GFP) into the mitochondrial matrix. Image acquisition was performed with an Axioskop2 fluorescence microscope (Carl Zeiss, Jena, Germany) equipped with a digital camera (micro-CCD).

### Statistical analysis

All data are the mean of three independent experiments. Bar error indicates standard deviation.

The number of stars (*) indicate the *p*-value range: * < 0.05, ** < 0.01, *** < 0.001.

## Results and discussion

To extend knowledge on favorable properties of the healthy properties of Melannurca Campana, we first studied the anti-ageing effects of the apple extract on the lifespan of the *Kllsm4Δ1* strain.

The extract composition was characterized by 63% procyanidin and 20% phloridzin with the addition of maltodextrin (See Methods).

**Fig. 1:**
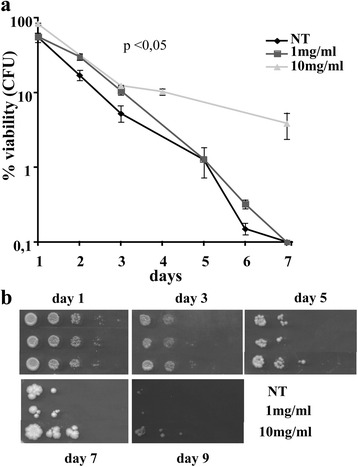
Apple extracts can prolong cellular viability during chronological aging. **a** Cellular viability of *Kllsm4*∆*1* in the presence of the indicated concentrations of apple extracts during ageing. Viability, measured over time, is expressed as a percentage of colony forming units. **b** Dilution spot assay of *Kllsm4*∆*1* strain at the indicated days in the absence (NT) or in the presence of the indicated concentrations of apple extracts. Plates were recorded after 3 days of incubation at 28 °C. *p*-value < 0.05

As shown in Fig. 1a, the viability of *Kllsm4Δ1* untreated cells significantly reduced during cultivation, as already known; in fact, after 2 days of growth the percentage of living cells was approximately 10% and the complete loss of viability was observed after 7 days of growth.

The presence of the apple extract at the concentration of 10 mg/ml clearly prevented cell death while lower doses of the extract (1 mg/ml) were almost ineffective.

We also performed a viability spot assay to compare the rate of cell growth under different conditions. The assay is based on serial dilutions of yeast cultures followed by plating cell samples on rich medium containing glucose as carbon source (YPD). As seen in Fig. 1b, after 9 days of growth at 28 °C cultures grown in the presence of 10 mg/ml apple extract showed cell survival up to the third dilution. In contrast, cells grown in the presence of 1 mg/ml extract or in the absence of the extract produced only few colonies after the same cultivation period.

It is known that reactive oxygen species (ROS) are the main cause of cellular ageing because of their high property of damaging DNA, proteins and lipids. Therefore, we checked the level of ROS in untreated and apple extract-treated yeast cells by using the dihydrorhodamine (DHR) 123 staining method (See Methods).

**Fig. 2:**
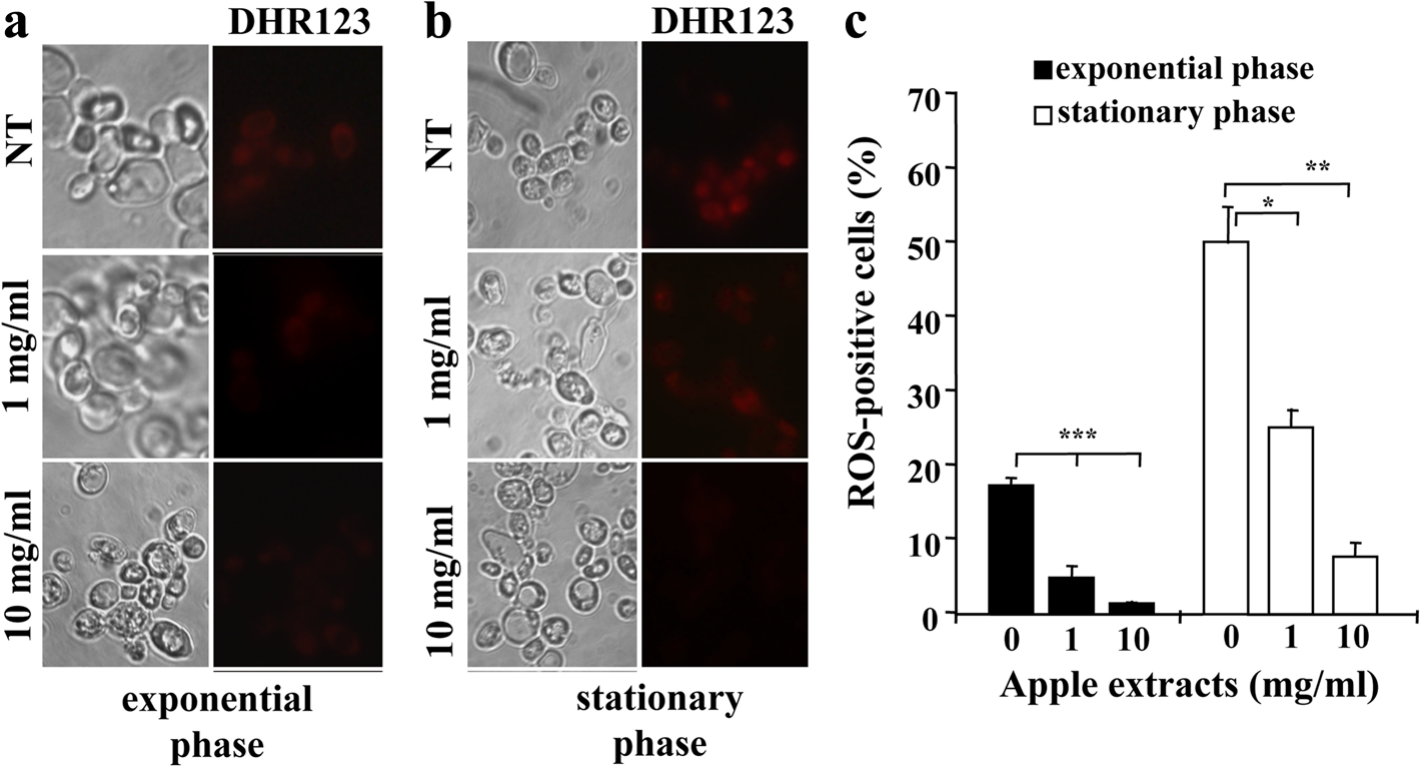
Apple extracts reduce cellular ROS levels. Dihydrorhodamine 123 (DHR123) staining of the *Kllsm4*∆*1* cells during exponential (**a**) and stationary (**b**) phases in the absence (NT) and in the presence of apple extracts at the indicated concentrations. The left column represents phase contrast relative to DHR123 staining. Panel **c** represents the average percentage of ROS positive cells from three independent experiments. *p*-value: * < 0.05; ** < 0.01; *** < 0.001

We previously reported that, in the absence of external oxidative stress, *Kllsm4Δ1* cells accumulate ROS during exponential and stationary phase [14]. As shown in Fig. 2, in the presence of apple extracts the ROS level was significantly reduced in both exponential (dividing cells, Fig. 2a) and stationary phases (resting cells, Fig. 2b). In particular, after treatment with 1 mg/ml apple extract, the percent of ROS-positive cells dropped from 17.2 to 4.7% during the exponential phase and from 50.2 to 25.3% after 3 days of growth. A similar result was obtained with the apple extracts at the concentrations of 2 mg/ml and 3 mg/ml (data not shown). We observed better results using the concentration of 10 mg/ml that allowed a reduction of ROS-positive cells to 1.3 and 7.8% during the exponential and the stationary phase, respectively.

Using a viability assay, we also tested the protective effect of the apple extract on the survival of yeast cells exposed to oxidative stress with hydrogen peroxide.

**Fig. 3:**
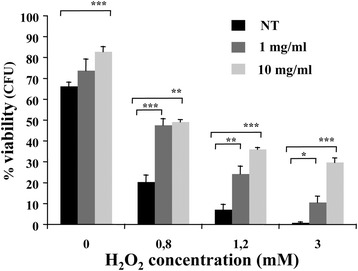
Cell viability of *Kllsm4*∆*1* was measured after exposure to H_2_O_2_ at the indicated concentrations for 4 h without (NT) or with apple extracts. Average of three independent experiments and standard deviation is reported. *p*-value: * < 0.05; ** < 0.01; *** < 0.001

As shown in Fig. 3, both in the presence of 1 mg/ml and 10 mg/ml of apple extracts, cells showed a higher viability compared to the control cultures.

Increasing the H_2_O_2_ concentration up to 3 mM, the best protective effect was observed at 10 mg/ml of extract, which resulted in 29.8% of cell survival, compared to about 11 and 0.9% survived cells in suspensions with the 1 mg/ml extract and control group, respectively. Cell viability increased at both concentrations also in the absence of H_2_O_2_, indicating that apple extracts might exert stimulating effects on cell proliferation under normal normal conditions.

These results demonstrated that apple extracts show dose-dependent antioxidant activities that play an important role in preventing cell damages from oxidative stress.

**Fig. 4:**
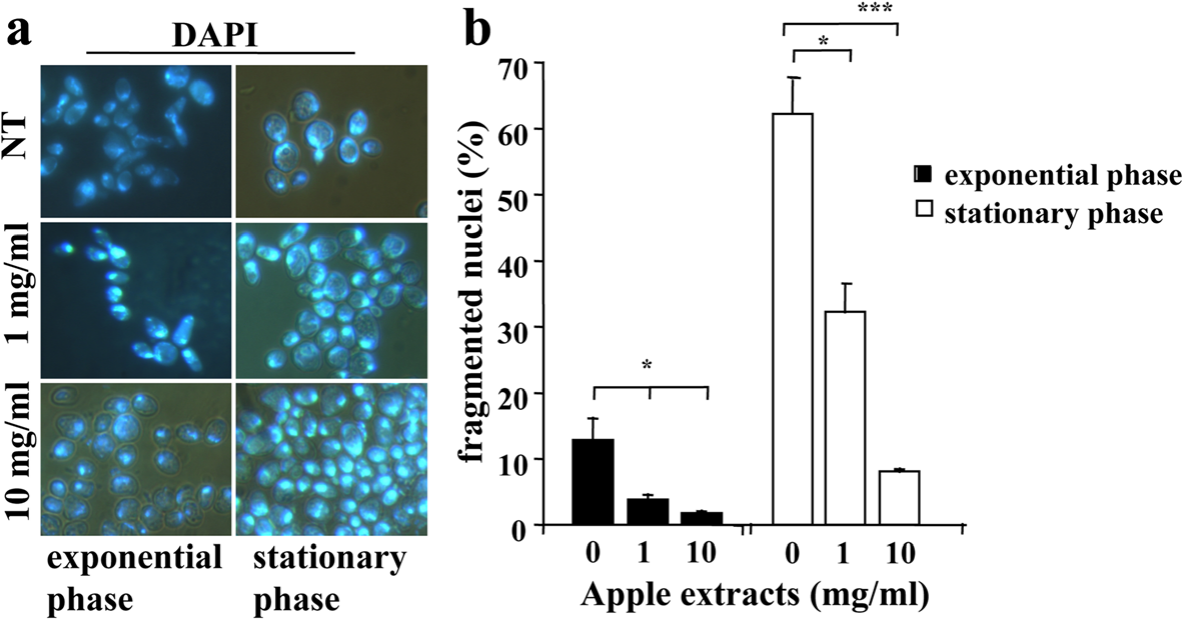
DAPI staining of the *Kllsm4*∆*1* cells during exponential and stationary phases (**a**) in the absence (NT) and in the presence of apple extracts at the indicated concentrations. (**b**) represents the percentage of fragmented nuclei in three independent experiments. *p*-value: * < 0.05; *** < 0.001

In addition to the shortened lifespan, the *Kllsm4Δ1* mutant shows apoptotic death hallmarks, such as nuclei and mitochondria fragmentation [15]. For this reason, we asked the question if the apple extracts could recover these phenotypes and prevent the premature cell death. We first assayed the nuclear fragmentation by staining cell nuclei with the fluorescent dye DAPI. As shown in Fig. 4, we observed a decrease in the percentage of fragmented nuclei during both exponential and stationary phase. In particular, during the stationary phase (3 days of growth) the number of cells with fragmented nuclei dropped from 62.3% (untreated cells) to 32.3 and 8.1% in presence of 1 mg/ml and 10 mg/ml extracts, respectively.

These results are in agreement with the observation that apple pectin can reduce apoptosis in a rat model of ischemia/reperfusion by inhibition of caspase and increase of Bcl-2/Bax [22].

**Fig. 5:**
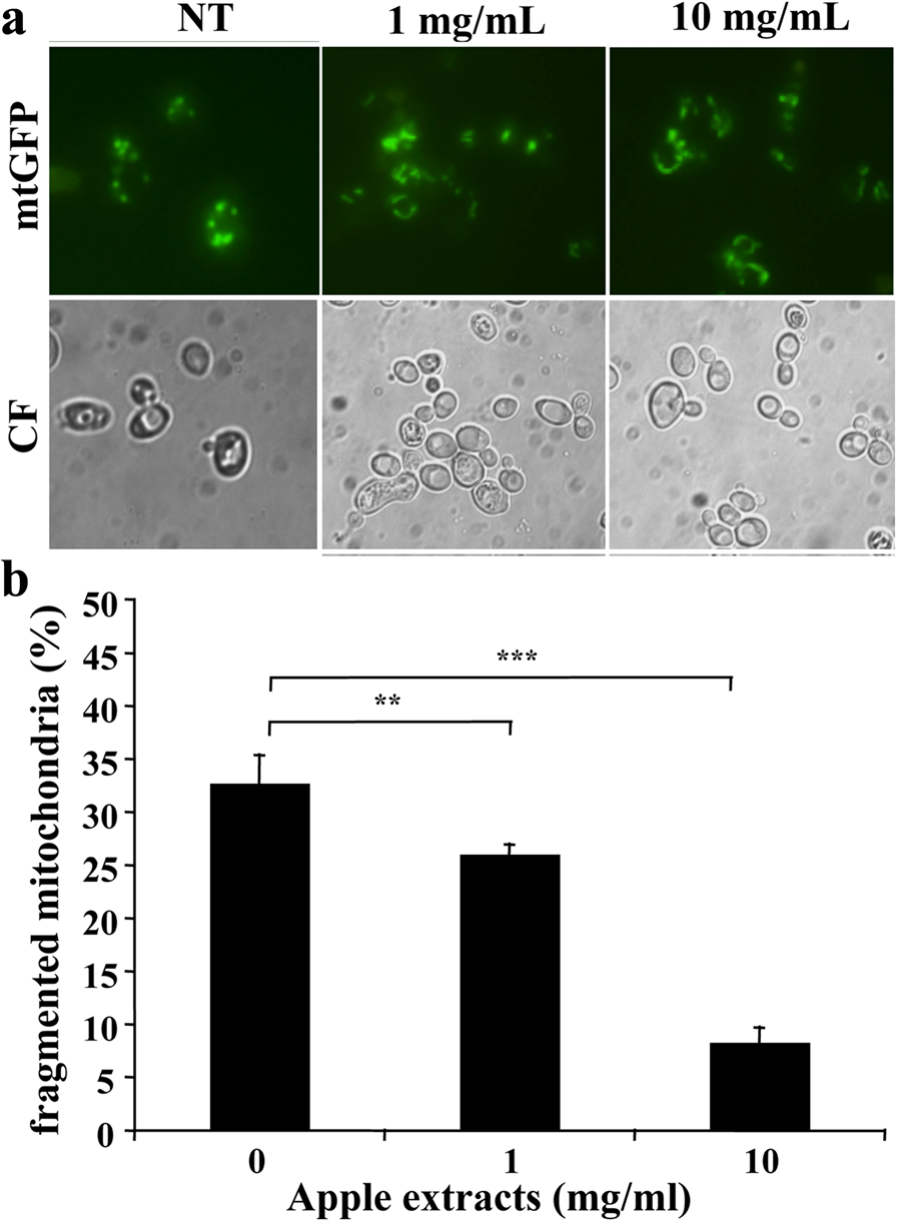
**a** The *Kllsm4*∆*1 S. cerevisiae* strain was transformed with mito-GFP, which targets GFP into the mitochondrial matrix. Cells were grown in glucose (*SD*) in the absence (NT) and in the presence of apple extracts at the indicated concentrations. The mitochondria morphology was evaluated by analyzing the GFP with an Axioskop2 fluorescence microscope (Carl Zeiss, Jena, Germany) equipped with a digital camera (micro-charge-coupled device). **b** Quantification of cell population showing fragmented mitochondria. Data represent the mean of three independent experiments. *p*-value: ** < 0.01; *** < 0.001

It is also known that, from yeast to mammals, mitochondria play a pivotal role in determining cell ageing and apoptosis [23, 24]. In fact, pro-apoptogenic factors (cytochrome c, Aif, EndoG) stored within mitochondria are released in the cytoplasm upon apoptotic stimuli. In addition, the normal mitochondrial tubular network is disrupted by such stimuli, as well as during cellular ageing [25]. We previously reported that a large part of *Kllsm4Δ1* cells presents fragmented mitochondria [15] and therefore we analysed the mitochondrial morphology in this mutant after treatment with apple extracts. As reported in Fig. 5, the apple extract could recover, at least in part, the mitochondria integrity. In particular, at the concentration of 10 mg/ml, the number of cells showing fragmented mitochondria was reduced to about 7% compared to 33% of the control.

## Conclusions

In this paper, we used the yeast *Saccharomyces cerevisiae* as a model to determine the anti-ageing and antioxidant properties of Annurca extracts. We found that the apple extracts can extend yeast lifespan and reduce ROS levels protecting cells from oxidative stress.

These findings confirmed our previously results with extracts from another sort of apple, the Golden Delicious produced in Trentino, Italy. This indicates that such properties seem to be essentially common to apple fruits.

In addition, since intracellular ROS are well known triggers of apoptosis, we also demonstrated that apple extracts strongly reduced the fragmentation of nuclei and of the mitochondrial network, which are considered main peculiar markers of apoptotic cell death. We do not know, at the moment, the relationships between these phenotypes and lifespan extension and many questions remain open concerning the identification of genes and mechanisms underlying the anti-ageing effects of apples.

Recently, using the replicative lifespan assay it has been reported that phloridzin, a major phenolic compound present in apple and apple juice, has anti-ageing effects on yeast by reducing the ROS level in cells exposed to oxidative stress. Interestingly, the transcription of *SOD1, SOD2* and *SIR2* genes, involved in the anti-oxidative stress and in the regulation of lifespan, respectively, increased after administration of phloridzin to the cells [26].

These facts and our results strongly suggest that apple extracts can extend cellular lifespan by lowering the level of intracellular ROS and by the activation of cellular signaling pathways modulating gene expression.

Future research should be focused on identification of responsive genes and pathways, and the availability of the various “omics” technologies developed in yeast can certainly be of help to this purpose.

## Abbreviations

DAPI: 4′,6-diamidino-2-phenylindole
DHR: Dihydrorhodamine 123
GFP: Green fluorescent protein
HDL: High-density lipoproteins
PGI: Protected geographical indication
ROS: Reactive oxygen species
SD: Synthetic dextrose
YPD: Yeast extract peptone dextrose
